# The Relationship Between Depressive Symptoms and Self-Neglect in Chinese Older Adults Living Alone: A Latent Profile Analysis

**DOI:** 10.3390/healthcare13060676

**Published:** 2025-03-20

**Authors:** Yali Hu, Miaomiao Wu, Yan Zhang, Lunfang Xie

**Affiliations:** 1 School of Nursing, Anhui Medical University, Hefei 230032, China; 18895376055@163.com (Y.H.); curew98@163.com (M.W.); 2 Department of Geriatric Respiratory and Critical Care Medicine, The First Affiliated Hospital of Anhui Medical University, Hefei 230022, China; 3 School of Health Service Management, Anhui Medical University, Hefei 230032, China; zhangymail@ahmu.edu.cn; 4 School of Nursing, The First Affiliated Hospital, Anhui Medical University, Hefei 230032, China

**Keywords:** older adults, living alone, depression, self-neglect, latent profile analysis

## Abstract

**Objectives**: To clarify the latent profile of depressive symptoms in Chinese older adults living alone and to explore the relationship between this profile and self-neglect. **Methods**: Data from the 2018 Chinese Longitudinal Healthy Longevity Survey (CLHLS) were utilized to conduct a latent profile analysis for the identification of depressive symptoms. Logistic regression was employed to analyze the related factors. Generalized linear modeling was used to assess the impact of different profiles of depressive symptoms on self-neglect. **Results**: A total of 1822 older adults living alone, with a mean age of (83.60 ± 9.15) years, were included in the study. Three categories of depression were identified: the C1 mild depression–sleep disturbance group (29.36%), the C2 moderate depression–forcefulness group (55.22%), and the C3 major depression–loneliness loss group (15.42%). Logistic regression analysis indicated that gender, place of residence, annual household income, educational level, reason for living alone, self-rated health status, cognitive function, and emotional support were significant influencing factors (*p* < 0.05). The risk of experiencing higher levels of self-neglect in the C2 was 1.264 times greater than in the C1. Furthermore, the risk of higher levels of self-neglect in the C3 was 2.040 times greater than in the C1. **Conclusions**: Heterogeneity in depressive symptoms is evident among Chinese older adults living alone, with variations in self-neglect across different potential categories of these individuals. The focus should be on older adults in the C2 and C3 profiles. This study proposes targeted intervention strategies from family, community, and policy development perspectives to help improve self-neglect in older adults.

## 1. Introduction

The World Health Organization suggested in 2024 that the pace of population aging is much faster than in the past [1]. China is recognized as one of the countries with the fastest-growing aging population. According to data from the seventh national population census conducted in 2020, the population aged 60 years and over was 264.02 million, constituting 18.7 percent of the country’s total population, while the elderly population aged 65 years and over made up 13.5 percent of the total national population [2]. The steady increase in the elderly population has made the issue of healthy aging a hot topic in society. Simultaneously, with the frequent movement and migration of the population, the family structure in China has gradually shifted from large to small. The number of single-generation households is increasing in both urban and rural areas, and the proportion of older adults living alone is also gradually increasing [3]. The number of elderly households living alone in China reached 37.29 million in 2020 [2]. The number of older adults living alone in China is rising, making them a social group that cannot be overlooked. With the dual effects of accelerated aging and significant population mobility, the number of elderly individuals experiencing psychological issues is also on the increase. Depressive symptoms are a prevalent and atypical mental health concern among the elderly population. The impact of living alone on depressive symptoms in older adults is becoming an important research question of interest to scholars. Byeon [4] stated in his study that the prevalence of depression among older adults living alone was 7.7%. Several studies have indicated a strong correlation between living alone and depression in older adults, with a higher depression index observed in those living alone compared to those who do not live alone or empty nesters. Additionally, there are higher rates of depressive symptoms and depression detection, as well as a greater risk of suicide among the elderly living alone [5,6]. At the same time, geriatric depressive symptoms are associated with a variety of complications, such as cognitive dysfunction [7], insomnia, dyspepsia, constipation [8], and so on, which seriously affect their quality of life.

Elderly neglect encompasses self-neglect, family neglect, and social neglect [9]. Self-neglect refers to behaviors that pose a threat to the health and safety of older adults, which are typically persistent and unintentional [10]. It is primarily characterized by issues in personal life, living environment, and social interactions, such as the neglect of safety, medical care, and interpersonal relationships. Self-neglect can be categorized into four dimensions: mode of life neglect, social communication neglect, medical care neglect, and living condition neglect [9]. Self-neglect can lead to an abnormal life status, increase the risk of hospital readmission and death, and is strongly associated with disability, debility, and suicidal ideation in older patients. Self-neglect among older adults has emerged as a serious and widespread social health issue [11]. A 12-year study in Chicago revealed that 16.6% of community-dwelling older adults experienced self-neglect [12]. A study in South Korea revealed that 22.8% of older adults living alone exhibited self-neglect [13]. In Poland, the rate of self-neglect among older adults was 11.4% [14]. In China, the prevalence of self-neglect among older adults is also concerning. A meta-analysis revealed that approximately 18.0% of older adults in China exhibit signs of self-neglect [15]. The limited social interaction experienced by elderly individuals living alone, such as the absence of a spouse and infrequent contact with children or other relatives and friends, heightens the risk of self-neglect. Yi’s study indicated that the screening rate for self-neglect among older adults living alone was 33.9% [16].

Research has shown that factors such as age, literacy, economic status, physical health, psychological status, and family support influence self-neglect in older adults. Additionally, several studies have shown that depression is strongly associated with self-neglect in older adults. Depression may predispose older people to poor lifestyles, make self-care more complex, and lead to self-neglect. Hansen [17] found that self-neglecting older adults had a depression detection rate ranging from 51% to 62%. Burnet [18] utilized propensity matching and determined that over 50% of self-neglecters experienced at least mild depression, a rate significantly higher than that of the matched non-self-neglect group. Li [19] indicated that self-neglect was more prevalent among older adults experiencing depression, and their self-neglect was particularly concerning. Yu [20] demonstrated that depression played a significant role in self-neglect. As for the causal relationship between self-neglect and depression, the academic community generally considers depression to be a predictor of self-neglect in older adults [21,22]. This is because depression can lead to emotional apathy, a loss of interest, and neglect of daily needs, health issues, and social interactions. Additionally, individuals with depression often have a negative attitude towards external supportive services, such as community services and healthcare, viewing them as unhelpful [23]. A review of the literature revealed that previous studies on depression in elderly people living alone primarily differentiated their level of depression based on scale scores, assuming homogeneity among all samples while overlooking the fact that “even individuals assessed as having the same level of depression may have qualitative differences” [24]. That is, there may be potential categories of older adults living alone at the same level of depression, and there may be differences in the relationship between different categories of depression and self-neglect. Previous research tentatively confirmed the relationship between depression and self-neglect, but the relationship between different categories of depression and self-neglect is unclear. Based on this, the study proposes the following hypothesis: among elderly individuals living alone who are at the same level of depression, there exist potential categories, and there are differences in the relationship between depression and self-neglect across these different categories.

Thus, utilizing data from the 2018 Chinese Longitudinal Healthy Longevity Survey (CLHLS), the study’s purpose was to analyze the heterogeneity of depression among Chinese older adults living alone and to explore differences in total self-neglect scores and dimensions among older adults in different depression categories. The study facilitates the implementation of categorical interventions to provide a basis for individualized interventions for older people living alone in different depressive categories. Also, it fills a research gap between the heterogeneity of depression and the relationship with self-neglect.

## 2. Materials and Methods

### 2.1. Data Sources and Sample Selection

**Figure 1:**
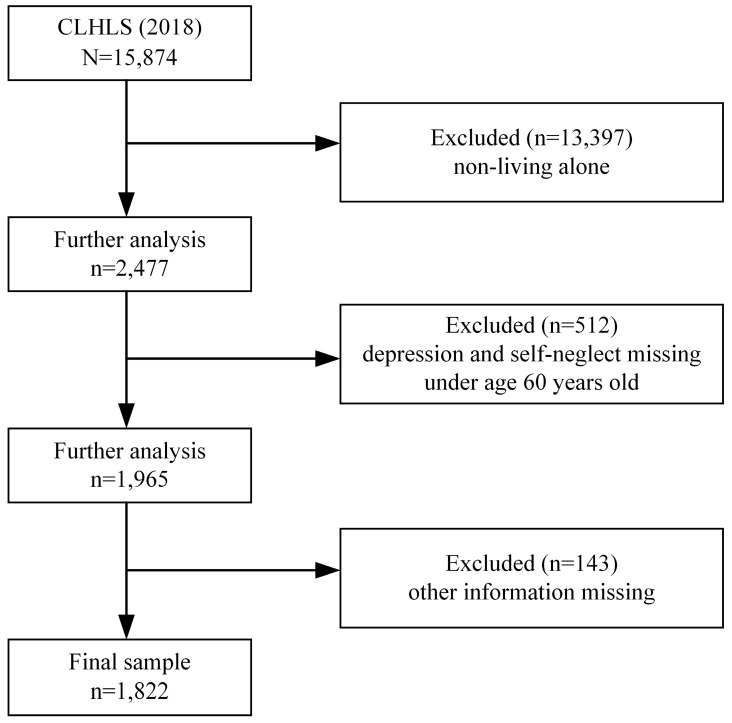
Flowchart for screening the study sample.

This study utilizes data from the 2018 Chinese Longitudinal Healthy Longevity Survey (CLHLS), a tracking survey on health determinants in old age conducted by the Center for Healthy Aging and Development Research at Peking University. The CLHLS database is the most extensive and longest-running social science survey in China, employing a multi-stage, unequally proportioned targeted random sampling method. It covers 23 provinces, municipalities, and autonomous regions nationwide. The survey is scientifically rigorous and nationally representative, with a sample response rate of about 90% [25]. The survey encompassed the fundamental aspects of older adults and their families, including socio-economic background, family structure, financial resources, economic status, self-assessment of health and quality of life, cognitive function, personality and psychological traits, daily living abilities, lifestyle, life care, and the bearing of medical expenses. The program conducted seven follow-up surveys after the baseline survey 1998, with the most recent follow-up survey (2017–2018) interviewing 15,874 older adults. According to the purpose of the study, the participants under the age of 60, not living alone and with missing or incomplete data on depression and self-neglect, were excluded. Of the 1965 participants who met the study criteria, 143, with more than 10% missing information on other variables, were excluded, resulting in 1822 valid responses and a response rate of 92.72% for this study. Multiple imputation was used to fill in the missing covariates, with default linear regression imputation for continuous variables and logistic regression for categorical variables, with five imputations performed. Finally, the imputed datasets were pooled and merged. The screening process is shown in Figure 1.

### 2.2. Variables

#### 2.2.1. Self-Neglect

Referencing previous scholarly studies [9,26], the questionnaire items were selected based on the following four dimensions of the self-neglect concept: mode of life, social communication, medical care, and living conditions. Mode of life includes regular consumption of fresh fruits and vegetables and regular exercise. Social communication refers to participation in social activities. Medical care encompasses timely hospital access for treating serious illnesses in older adults and annual routine medical check-ups. The living conditions include any instances of rain leaks, flooding, or broken pipes in the home over the past year, as well as the presence of a constant musty smell. Responses to all items were coded as “yes” or “no”. Mode of life, social communication, and medical care options were scored as “yes = 0” and “no = 1”, while the living conditions were scored as “yes = 1” and “no = 0”. The total score could reach 8, with higher scores indicating greater self-neglect in older individuals.

#### 2.2.2. Depression

Depressive symptoms among older adults were assessed using the Centre for Epidemiological Studies Brief Depression Scale (CES-D), which comprises 10 items. The database includes questions such as the following: ‘Do you get annoyed by small things?’ and ‘Are you having trouble concentrating on things right now?’ ‘Do you feel sad or depressed?’ ‘Do you feel that the older you get, the less useful you are and have difficulty doing anything?’ ‘Do you feel nervous or scared?’ ‘Do you feel lonely?’ and ‘Do you feel unable to get on with your life?’ Respondents can answer with ‘never’, ‘rarely’, ‘sometimes’, ‘often’, or ‘always’, in descending order of severity. For the questions ‘Are you hopeful for the future?’ ‘Do you feel as happy as you were when younger?’ and ‘How well do you sleep now?’ the reverse assignment is applied. The total score ranges from 10 to 50, with higher scores indicating greater levels of depression in older adults. The Cronbach’s α for the depression scale in this study was 0.817.

#### 2.2.3. Cognitive Function

The cognitive functioning of older adults was evaluated using a revised version of the Brief Mental State Examination (MMSE), which comprises 24 items across five domains: general ability, orientation, attention and calculation, recall, and language and visuospatial skills. For each of the 23 items, 1 point was given for a correct response and 0 points for an incorrect one, whereas the remaining item, ‘Tell me what you can eat in one minute’, was scored with 1 point for one answer and 7 points for seven or more answers. The total score ranged from 0 to 30. Cognitive dysfunction was indicated when the total score was less than 24, and normal cognitive function was indicated when the total score was 24 or higher. The Cronbach’s α for the depression scale was 0.910 [27].

#### 2.2.4. Activities of Daily Living

The Katz scale was used to assess the ability of older adults to perform daily activities across six categories: bathing, dressing, toileting, indoor walking, bowel control, and eating. The results for each category were categorized as ‘no difficulty’, ‘some difficulty’, or ‘unable to perform’ based on the participants’ actual situations. Each daily activity scored 1 point for inability to complete, 2 points for some difficulty, and 3 points for no difficulty. The total score range for the six activities was 6 to 18 points, with higher scores indicating a better ability to perform daily activities among older adults. The Cronbach’s α for this scale in this study was 0.745.

#### 2.2.5. Socio-Demographic Factors

The following variables were selected and assigned values based on the socio-demographic characteristics of the study population: Age (60–74 years = 1, 75–89 years = 2, ≥90 years = 3), sex (male = 1, female = 2), place of residence (dummy variable, rural = ‘first’, followed by city, town), reason for living alone (dummy variable, other = ‘first’, the absence of nearby children, avoid inconveniencing their children), self-rated health status (very good = 1, better = 2, fair = 3, worse = 4, very bad = 5), years of education (dummy variable, ≥7 years = ‘first’, followed by 0 years, 1 to 6 years), source of livelihood (dummy variable, pension = ‘first’, followed by family, other), annual household income (dummy variable, ≥100,000 RMB = ‘first’, followed by <30,000 RMB, 30,000 to 49,999 RMB, 50,000 to 99,999 RMB), marital status (dummy variable, married = ‘first’, followed by unmarried/divorced, widowed), and distance to a healthcare facility (<5 km = 1, ≥5 km = 2). The number of emotional support items was recorded as continuous variables.

### 2.3. Methodological Rationale

The Latent Class Model (LCM) is an individual-centered analytical technique. Its individual-centered orientation aids in clarifying inconsistent conclusions from previous studies, identifying specific conditions for the validity of a theory based on latent classes, and, notably, exploring the relationship between each class and the antecedent and outcome variables. Variables are categorized into two types: categorical variables, which are analyzed through Latent Class Analysis (LCA), and continuous variables, which are analyzed through Latent Profile Analysis (LPA) [28]. The LCM has been widely used in various fields, including sociology, biomedicine, and psychology, due to its excellence in heterogeneous group categorization studies [29]. By employing a categorization approach, the model aims to place elderly people living alone who exhibit similar response patterns to depressive symptoms into the same potential category. This approach helps us to analyze in depth the unique depressive characteristics of each potential category group and to paint a clear picture of the distribution of older adults living alone across depressive categories. The LCM also helps to capture group heterogeneity, which is difficult to observe in variable-centered studies, and which is essential for gaining insights into the diversity and individual differences in depressive symptoms among older adults living alone. Consequently, the main objective of this research is to employ an individual-centered approach to categorize depression among Chinese older adults living alone and to examine the variations in overall self-neglect scores, as well as across different dimensions, among these older adults based on their depression classifications.

### 2.4. Statistical Analysis

#### 2.4.1. Descriptive Analysis

The collected data were analyzed using descriptive statistics, where the measures were described using mean and standard deviation if they satisfied a normal distribution and median and interquartile range if they did not. The counts were described using constituent ratios and rates.

#### 2.4.2. Potential Profile Analysis

Potential profiling of depression scores for each entry was performed using Mplus 8.3, starting with an initial model and progressively increasing the number of categories until the model that best fits the data was found. Fit information includes metrics such as Akaike information criterion (AIC), Bayesian information criterion (BIC), Sample size adjusted BIC (aBIC), Lo–Mendell–Rubin likelihood ratio test (LMRT), Bootstrapped likelihood ratio test (BLRT), Entropy, and average posterior probability (APP) for each category, with lower AIC, BIC, and aBIC values and higher Entropy values, as well as LMRT and BLRT reaching significant fit information, being preferred. When the AIC and BIC values are consistently monotonically decreasing, selecting the model corresponding to the inflection point in the decreasing trend is preferable. The Entropy value should be greater than 0.60, and when the Entropy value is greater than 0.8 or higher, it indicates that at least 90% of the individuals are correctly classified; the probability of belonging for each mean should be greater than 0.70. Models with a sample size less than 5% of the categories are considered meaningless and should be discarded. The interpretability of the results also needs to be considered to determine the best profile model.

#### 2.4.3. One-Way Analysis of Variance

The general characteristics, emotional support, and other factors of older adults living alone were compared across depression potential profiles using chi-square tests, Analysis of variance (ANOVA), or non-parametric tests. ANOVA and *t*-tests assessed differences in self-neglect scores among older adults based on demographic characteristics. Spearman’s correlation analysis was conducted to examine the relationship between depression, activities of daily living, self-rated health status, and self-neglect scores in older adults living alone. To evaluate between-group differences in depression on the dimensions of self-neglect, the non-parametric Kruskal–Wallis test was applied to compare the mean rank order of the groups, as the data did not meet the criteria for normal distribution.

#### 2.4.4. Multifactorial Analysis

Logistic regression analyses were conducted to explore the predictors of the potential profile of depression in older adults, using the potential profile of depression as the dependent variable and the candidate predictor variables (demographics, health, and factors related to living alone) as the independent variables. An unordered multinomial logistic regression was used because the parallel lines test was not satisfied.

Generalized linear modeling was employed to investigate the association between latent profiles of depressive symptoms and self-neglect, with self-neglect in older adults living alone serving as the dependent variable and indicators that were statistically significant in univariate analyses as independent variables.

## 3. Results

### 3.1. General Characteristics of Older Adults Living Alone

The study included 1822 older adults living alone, with a mean age of (83.60 ± 9.15) years—1120 (61.47%) were female, and 702 (38.53%) were male. Regarding residence, 957 (52.52%) of the older adults lived in city and town, while 865 (47.48%) resided in rural areas. Nearly half (54.28%) of the older adults had no formal education. A significant majority, 1582 (86.83%), were widowed. Regarding financial dependence, 831 (45.61%) relied on family members, 441 (24.20%) on pensions, and 580 (31.83%) on other sources of income. Moreover, more than half (71.13%) of the older adults had an annual household income of less than RMB 30,000 (USD 4110).

Of the participants, 1591 (87.32%) reported above-average self-rated health, and 259 (14.22%) exhibited cognitive dysfunction. Regarding living arrangements, 1245 (68.33%) of older adults lived alone by choice, to avoid inconveniencing their children, 413 (22.67%) lived alone because of the absence of nearby children, and 164 (9.00%) had other reasons for living alone. Details are shown in Supplementary Materials: Table S1.

### 3.2. Potential Profiles of Depressive Symptoms in Older Adults Living Alone

This study utilized the 10 items of the CES-D10 scale as continuous exogenous variables to explore six potential categorical models, as depicted in Table 1. As the number of model categories increased incrementally, the AIC, BIC, and aBIC values decreased. Entropy was >0.8 in categories 3 to 6, and LMR > 0.05 in category 4, indicating that category 4 was not superior to category 3. Taking into account the practical significance, interpretability, and simplicity of the model and classification outcomes, along with the minimum proportion of each profile, the three-category model was deemed the optimal fit.

**Figure 2:**
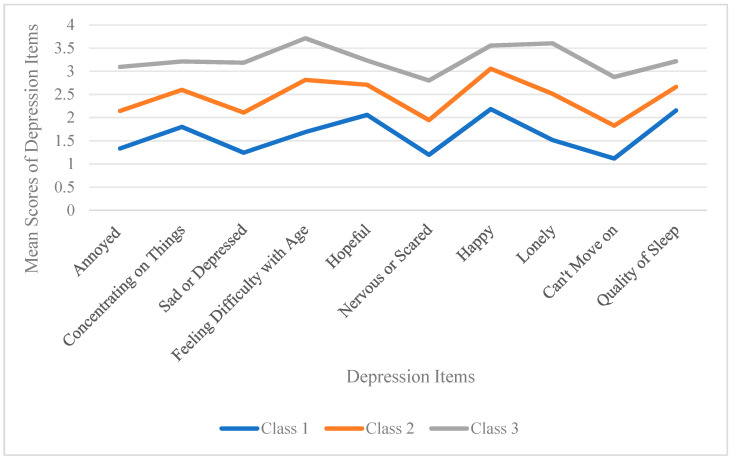
Potential profiles of depressive symptoms in Chinese older adults living alone.

Depressive symptoms among Chinese older adults living alone were classified into three categories through profile analysis, as shown in Figure 2. In group C1, there were 535 cases (29.36%), and the mean score for each item ranged from 1.12 to 2.18, with a total score of 16.29 ± 4.08. The score for the 10th item, ‘How is the quality of your sleep?’ was notably high, leading to the group being named the ‘mild depression–sleep disturbance group’. In group C2, there were 1006 cases (55.22%), the mean score for each item ranged from 1.83 to 3.06, and the total mean score was 24.38 ± 4.07. High scores were observed in the seventh item, ‘Do you feel as happy as when you were young?’, the fourth item, ‘Do you feel less useful as you get older?’, and the second item, ‘Is it hard for you to concentrate on your work now?’. Due to these relatively high scores, the group was named the ‘moderate depression–forcefulness group’. In group C3, there were 281 cases (15.42%), the mean score for each item ranged from 2.80 to 3.60, and the total mean score was 32.48 ± 2.99. High scores were also seen in the fourth item, ‘Do you feel that the older you get, the more useless you feel and that it is hard for you to do anything?’ and the eighth item, ‘Do you feel lonely?’ Consequently, the group was named the ‘major depression–loneliness loss group.

### 3.3. Analysis of Factors Influencing Potential Profiles of Depressive Symptoms in Older Adults Living Alone

The results of the logistic regression are presented in Table 2. The analysis indicated that gender, place of residence, years of education, annual household income, reason for living alone, health status, cognitive function, and emotional support are valid predictor variables for the latent profile of depression. When comparing the C1 mild depression–sleep disturbance group with the C2 moderate depression–forcefulness group, the likelihood of belonging to the latter was higher for individuals living in town (OR = 1.341, 95% CI = 1.037–1.733), with an annual household income of less than RMB 30,000 (USD 4110) (OR = 1.682, 95% CI = 1.144–2.472), living alone because of the absence of nearby children (OR = 1.697, 95% CI = 1.098–2.623), and those living alone to avoid inconveniencing their children (OR = 1.554, 95% CI = 1.061–2.276). Additionally, individuals with poorer physical health were more likely to be classified into the C2 moderate depression–forcefulness group (OR = 2.066, 95% CI = 1.794–2.379).

When comparing the C1 mild depression–sleep disturbance group to the C3 major depression–loneliness loss group, females (OR = 1.451, 95% CI = 1.009–2.089), those who lived in town (OR = 1.730, 95% CI = 1.211–2.471), individuals without education (OR = 2.012, 95% CI = 1.038–3.901), with an annual household income of less than RMB 30,000 (USD 4110) (OR = 2.028, 95% CI = 1.049–3.919), those in poorer physical health (OR = 5.408, 95% CI = 4.336–6.747), experiencing cognitive dysfunction (OR = 1.962, 95% CI = 1.243–3.096), and those with low emotional support (OR = 0.826, 95% CI = 0.693–0.984) were more likely to belong to the C3 major depression–loneliness loss group.

When comparing the C2 moderate depression–forcefulness group with the C3 major depression–loneliness loss group, it was observed that older adults with poorer physical health (OR = 2.618, 95% CI = 2.164–3.166), cognitive dysfunction (OR = 1.509, 95% CI = 1.050–2.169), and less emotional support (OR = 0.732, 95% CI = 0.629–0.853) were more likely to belong to the C3 major depression–loneliness loss group.

### 3.4. Levels of Self-Neglect in Older Adults Living Alone

In the present study, the self-neglect score of older adults living alone was (2.98 ± 1.39), as shown in Table 3. Differences in self-neglect levels among older adults with varying demographic characteristics were compared, and the results indicated a significant difference (*p* < 0.05), except for gender and distance from a healthcare facility. Table 4 presents a correlation matrix plot between depression and self-neglect.

### 3.5. The Relationship Between Underlying Profiles of Depression and Self-Neglect in Older Adults Living Alone

**Figure 3:**
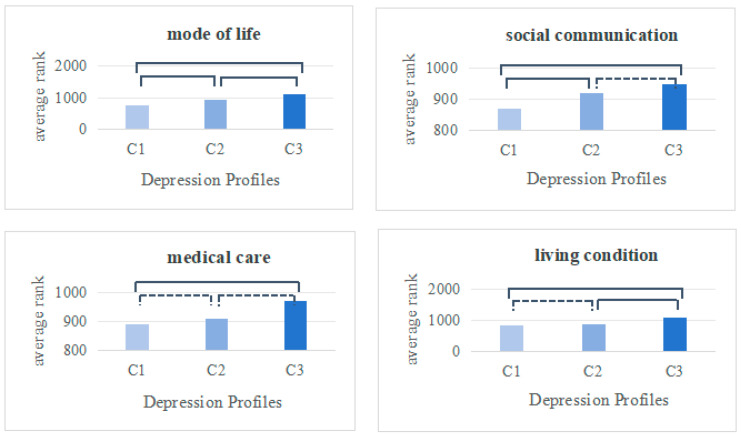
Comparison of potential profiles of depressive symptoms across dimensions of self-neglect. Note: C1 represents the mild depression–sleep disturbance group, C2 represents the moderated depression–forcefulness group, and C3 represents the majordepression–loneliness loss group; solid lines represent *p* < 0.05 and dashed lines represent *p* > 0.05.

The self-neglect scores for the C1 mild depression–sleep disturbance group, C2 moderate depression–forcefulness group, and C3 major depression–loneliness loss group of older adults living alone were (2.55 ± 1.37), (2.99 ± 1.31), and (3.77 ± 1.38), respectively. The scores among the three groups differed significantly (F = 75.398, *p* < 0.001). Additionally, the differences in scores across the four dimensions of self-neglect among the various depression profile groups were also statistically significant (*p* < 0.05). Furthermore, comparing the four dimensions of self-neglect between groups revealed the following results, as depicted in Figure 3: The C2 moderate depression–forcefulness group scored higher on the mode of life and social communication dimensions than the C1 mild depression–sleep disturbance group (*p* < 0.05). The C3 major depression–loneliness loss group exhibited higher scores than the C1 mild depression–sleep disturbance group across all four dimensions of mode of life, social communication, health care, and living condition (*p* < 0.05) and also scored higher than the C2 moderate depression–forcefulness group in the dimensions of mode of life and living condition (*p* < 0.05).

A generalized linear model was used with self-neglect as the dependent variable and the latent profile of depression in older adults living alone as the independent variable (with a dummy variable and the C1 mild depression–sleep disturbance group as the reference group) after controlling for confounding factors such as age, place of residence, years of education, source of livelihood, and cognitive function. Variables with meaningful results are presented. The results indicated that depression independently influenced self-neglect, with the C2 moderated depression–forcefulness group having a 1.264-fold increased risk of higher levels of self-neglect compared to the C1 mild depression–sleep disturbance group. Additionally, the C3 major depression–loneliness loss group exhibited a 2.040-fold increased risk of higher levels of self-neglect compared to the C1 mild depression–sleep disturbance group (all *p* < 0.001). These findings are detailed in Table 5.

## 4. Discussion

Previous research on depressive symptoms and self-neglect among elderly individuals living alone primarily categorized their levels of depression based on scale scores. This approach assumes homogeneity across all samples yet overlooks the reality that “even individuals assessed as having the same level of depression may exhibit qualitative differences”. The current study investigated whether there are variations in self-neglect among older adults living alone who have distinct depressive profiles. It employed an individual-centered latent profile analysis to minimize internal differences within subgroups and maximize distinctions between subgroups. The present study found that depressed mood in older adults living alone has a distinct category profile and is characterized by group heterogeneity. The LPA analysis showed that the depressed mood of older adults living alone corresponds to three different profile types. According to the characteristics of each category, these three subgroups can be named as C1 mild depression–sleep disturbance group, C2 moderated depression–forcefulness group, and C3 major depression–loneliness loss group, respectively. Gender, place of residence, years of education, annual household income, reason for living alone, self-rated health, cognitive function, and emotional support were influential factors in the latent profile of depressive symptoms in older adults.

The percentage of 29.36% of older adults were categorized in the C1 mild depression–sleep disturbance group. The item “How well do you sleep now” scored relatively high compared to the other items. This may be related to the fact that under the influence of deteriorating organ function, this group of older adults is prone to sleep disorders such as difficulty falling asleep and waking up often during the night. Previous research confirmed that there is a bidirectional causal relationship between sleep disorders and depression: Chronic sleep disorders may exacerbate depression in older adults, and depression itself can interfere with sleep patterns, creating a vicious cycle [30].

The percentage of 55.22% of older adults were categorized in the C2 moderated depression–forcefulness group, which scored relatively high on the items “Not as happy as when I was younger”, “struggling to do things”, and “difficulty concentrating”. These items scored high and were dominated by somatic symptoms. Compared to individuals in the C1 and C3 groups, older adults residing in towns, with low household incomes, poor self-rated health, and living alone because of the absence of nearby children, tend to avoid inconveniencing their offspring and are more likely to belong to this group. The prevalence of depression among older adults residing in towns is comparable to the findings of Chen et al. [31]. China exhibits an urban–rural dual structure, where towns act as transitional zones between urban and rural areas. The uneven economic and social development among cities, towns, and villages has resulted in disparities in older adults’ lifestyles, retirement patterns, and retirement resources. Wu observed that older individuals residing in towns have a greater demand for elderly services than those in rural and urban areas [32]. Due to their unique living style, older adults living alone are susceptible to numerous psychological and physiological issues. When the needs of these individuals are unmet over an extended period, they are at risk of experiencing depression and loneliness. A favorable family economic situation offers older adults more resources and capabilities to cope; they can enjoy improved living conditions, possess adequate social capital, view problems from multiple perspectives, and understand more about mental health, thereby better managing their mental health issues [33]. The subjective willingness to live alone can be categorized into active and passive solitude. Older adults who live alone passively, often due to the absence of their children, are susceptible to depression because they receive fewer family resources and less support than those who choose to live alone actively. Although living alone to avoid burdening their children is considered active solitude, there is a fundamental difference between this and living alone by choice for peace and quiet or because their children are nearby. Compared to older adults who choose to live alone for peace or because their children are nearby, these older adults may opt to trouble their children as little as possible, due to the pressures of their children’s lives, the shifting focus of intergenerational relationships downward, and differing concepts of how generations should live together [34]. Consequently, they may suppress their own needs and emotions, increasing the risk of depression.

The percentage of 15.42% of older adults were categorized in the C3 major depression–loneliness loss group, characterized by higher overall scores on items, a predominance of mood symptoms in this group, and significantly higher scores on the item “Do you feel sad or depressed” than in the other two groups. Compared to individuals in the C1 and C2 groups, older adults who were female, lived in towns, had less education, lower household incomes, had poor self-rated health status, cognitive dysfunction, and low emotional support were more likely to belong to this group. The ease with which females can belong to this category may be related to the different personality traits of different genders, with females having higher emotional susceptibility and being less adept at regulating negative emotions than males [35]. Good health serves as a protective factor against depressive symptoms. Self-rated health status reflects older adults’ subjective perception of their health and is positively correlated with mental health status [36]. A meta-analysis [37] indicated that older adults with three or more chronic diseases had a 3.86 times greater risk of depression compared to those without chronic diseases. Older adults experiencing poor physical health and chronic conditions are more susceptible to negative emotional responses due to both physical and mental distress [38,39]. Research conducted by several scholars [40,41] showed that depressive symptoms are inversely correlated with cognitive function. Moreover, cognitive function can predict depressive symptoms [42]. Due to deteriorating physical functioning, diminished social engagement, and cognitive decline, older adults are susceptible to feelings of helplessness and a diminished sense of self-worth, which can contribute to depression. Emotional support provides psychological comfort and care to older adults living alone, alleviating loneliness and feelings of helplessness, and making them feel understood and accepted. This support helps to bolster the older adults’ sense of value and self-identity [38]. Conversely, a lack of emotional support may exacerbate the severity of depression in older adults.

In this study, the self-neglect score of the elderly living alone was (2.98 ± 1.39). It was higher than those found in Chang’s study on empty-nesters [26]. This might be due to the fact that empty nesters encompassed individuals who lived alone as well as those who resided with a spouse. Spouses could offer emotional support and companionship, thereby alleviating the feelings of loneliness and depression. After accounting for the confounding variables, depression emerged as an independent predictor of self-neglect. The risk of higher levels of self-neglect in the C2 moderated depression–forcefulness group was found to be 1.264 times greater than that of the C1 mild depression–sleep disturbances group. Similarly, the risk of higher levels of self-neglect in the C3 major depression–loneliness loss group was 2.040 times greater than that of the C1 group. In essence, among the older adults who were living alone, the more severe the depression, the greater was the risk of self-neglect. It was consistent with the results of previous studies [16,43].

In previous research, the primary focus was on exploring the ubiquity of the effects of depression on self-neglect, while neglecting potential variations across different dimensions of self-neglect among various depression categories. In light of this, the present study further analyzed the differences in self-neglect dimensions among different depression categories. The C2 moderated depression–forcefulness group exhibited higher scores for lifestyle and social communication neglect compared to the C1 mild depression–sleep disturbance group. This may be attributed to several factors: older adults in this group often live passively alone, face poor economic conditions, lack available family resources, suffer from poor physical health, and experience a decline in aging functions, making tasks increasingly laborious. Challenges with concentration and other physical symptoms further compound these issues, leading to a lack of self-care motivation and ability, and a diminished willingness to engage in social interactions, resulting in the neglect of lifestyle and social activities. Elderly individuals who adopt unhealthy lifestyles and reduce social interaction activities increase their health risks. Thus, the interventions for this group aim to understand potential health issues in older adults, alleviate physical symptoms, foster healthy lifestyle habits, and encourage social engagement among the elderly. Communities establish electronic health records for older adults, conduct comprehensive assessments, identify high-risk groups for chronic diseases early on, clarify health issues and analyze their causes, and develop health guidance programs. Additionally, they provide regular follow-up, rehabilitation, and comprehensive management for the elderly already suffering from chronic diseases, aiming to improve physical symptoms caused by the original disease. Moreover, engaging in activities, like board games, can enhance attention and concentration [44]. Breaking down daily tasks and encouraging their gradual completion can improve older adults’ executive functions, while appropriate combinations of cognitive function training, nutritional support, and comprehensive psychological interventions can alleviate older adults’ somatic symptoms [45]. Mobile health interventions such as smartphone apps [46], virtual reality [47], and wearable technology [48] can also be considered to alleviate depressive symptoms in older adults.

The C3 major depression–loneliness loss group exhibited higher levels of neglect in the mode of life, social communication, medical care, and living conditions compared to the C1 mild depression–sleep disturbance group. Scores on the mode of life and living condition dimensions were higher than in the C2 moderated depression–forcefulness group, which was the primary focus. This group, primarily consisting of uneducated older females with poor physical health and cognitive dysfunction, lacked adequate emotional support. As a result, these older adults experienced a reduced sense of self-worth as they aged, leading to feelings of depression, sadness, and a lack of interest. They often neglected their daily needs, health concerns, social communication, and even medical care. This can lead to a further decline in physical functioning, an increased risk of chronic disease morbidity, weakened social participation, and accelerated cognitive deterioration in older adults, ultimately resulting in a triple burden on individual health, family caregiving, and public health resources. Thus, this group should focus on establishing an intervention system for self-neglect among older adults, leveraging the synergistic mechanism of family support and social resources. Bolstering intergenerational support and encouraging children to live with their parents can fully leverage the family’s traditional role as a provider of elderly care, especially in situations where a comprehensive old-age security system has not yet been established. For children unable to live with their parents, it is important to provide adequate material and emotional support to elderly individuals living alone. This includes regular visits and care, ensuring the safety of their living environment, conducting age-appropriate renovations to their housing to eliminate safety hazards, and monitoring their health status. Services such as health check-ups, early disease screening, and chronic disease monitoring and management for the elderly should be strengthened. Second, raising awareness among older adults about self-neglect is crucial. Communities have established records for older adults living alone, regularly assessing their social and psychological conditions at home. High-risk groups for self-neglect are identified early, and these individuals are carefully and patiently instructed on the relevant knowledge to enhance their awareness of self-safety and the importance of self-health. Moreover, the early identification of high-risk groups needs to be enhanced. It is recommended that a multi-linked platform that integrates medical and community services be established to share databases of high-risk individuals, automatically identifying these groups. Expanded community-based services and profiles of elderly individuals living alone should be created, and the elderly should be visited regularly to evaluate their social and psychological well-being, thereby raising their awareness of self-safety and the significance of maintaining their health. Social support systems should also be bolstered by promoting community support networks and encouraging mutual assistance through neighborhood and community activities, especially at the community level.

## 5. Conclusions

This study identified the existence of three profiles of depressive symptoms among Chinese older adults living alone. It explored the differences in total self-neglect scores and dimensions among older adults in different depressive categories. Future studies could develop some effective community health programs to prevent self-neglect among different depressive groups. For example, the Community Residents Committee and Community Health Service Center can collaborate to create a community elderly health information sharing platform, which can realize intelligent diagnosis of depression categories, propose personalized intervention plans for C2 and C3 groups, with timely intervention, health monitoring, and individual-family-community data sharing. During the project implementation process, community healthcare providers should focus on health assessment and health management, the Community Residents Committee should focus on eliminating safety hazards in the living environment and organizing various social activities, and family members need to be educated to strengthen emotional support for elderly adults. From the perspective of policy-making, the government should pay more attention to psychosocial problems among Chinese older adults living alone, especially those in low-income families. Policy on special subsidies for seniors living alone, graded reductions/exemptions in medical costs, elderly health promotion, and social participation are expected to be issues in the future.

## 6. Strengths and Limitations

The strengths of this study are as follows: First, the data were derived from the Chinese Longitudinal Healthy Longevity Survey (CLHLS), the most expansive and extended persistent social science survey in China, with an adequate and representative sample size. Second, this study employed LPA to investigate potential profiles of depression among older adults living alone. It further analyzed differences in self-neglect between these groups to offer new insights into research on depressive symptoms in this demographic and to inspire innovative approaches for personalized interventions targeting self-neglect in older adults living alone.

The limitations of this study are as follows: First, this study used the 2018 data of this database, which is the latest issue of public data that can be accessed at present, but there is a lag for the present. We will continue to track the database updates in the future. Second, potential profile analysis has advantages in group categorization but limitations such as subjectivity and uncertainty. In the future, standard classification methods such as cluster analysis can be compared with LPA, providing more comprehensive classification information and further validating the advantages and applicability of potential profile analysis. Third, the study’s reliance on self-reported measures is limited by their inherent subjectivity. Future research could incorporate subjective scales and objective indicators to enhance accuracy and rigor, ensuring more reliable findings. Fourth, elderly adults with dementia were not excluded from assessments of cognitive function and activities of daily living. This group may have introduced bias into the study’s result. Future studies need to take this fully into account and exclude dementia elderly. Fifth, the data in this study were collected at one point in time, and causal inferences between variables could not be made. Future studies could use prospective cohort studies to clarify causal relationships between variables.

## Figures and Tables

**Table 1:** Fit indices for latent profile analysis of depressive symptoms in Chinese elderly living alone.

Model	AIC	BIC	aBIC	Entropy	*p*		Smallest Class (%)
					LMR	BLRT	
**1**	51,863.65	51,973.80	51,910.26	-	-	-	-
**2**	48,654.99	48,825.73	48,727.24	0.777	<0.001	<0.001	44.13
**3**	47,538.80	47,770.13	47,636.70	0.824	<0.001	<0.001	15.42
**4**	47,158.13	47,450.03	47,281.66	0.834	0.0826	<0.001	2.36
**5**	46,843.12	47,195.60	46,992.28	0.846	0.0449	<0.001	2.25
**6**	43,796.62	44,209.70	43,971.42	0.894	0.0001	<0.001	5.05

Note: AIC = Akaike information criterion; BIC = Bayesian information criterion; aBIC = Sample size adjusted BIC; LMR = Lo–Mendell–Rubin likelihood ratio test; BLRT: Bootstrapped likelihood ratio test.

**Table 2:** Logistic regression analysis of factors influencing potential profiles of depressive symptoms in older people living alone.

Variable	C2vsC1		C3vsC1		C3vsC2	
	OR (95%CI)	*p*	OR (95%CI)	*p*	OR (95%CI)	*p*
Gender (Ref: Female)	1.055 (0.830–1.342)	0.661	1.451 (1.009–2.089)	0.045	1.375 (0.997–1.897)	0.052
Residence (Ref: Rural)						
City	0.998 (0.705–1.411)	0.989	0.619 (0.342–1.121)	0.114	0.621 (0.360–1.070)	0.086
Town	1.341 (1.037–1.733)	0.025	1.730 (1.211–2.471)	0.003	1.291 (0.954–1.746)	0.098
Years of education						
(Ref: ≥7)						
0	1.153 (0.795–1.672)	0.452	2.012 (1.038–3.901)	0.038	1.745 (0.948–3.212)	0.074
1–6	1.039 (0.724–1.490)	0.837	1.672 (0.869–3.216)	0.124	1.610 (0.878–2.952)	0.124
Annual family income						
(Ref: ≥100,000 RMB) (USD 13,702)						
<30,000 RMB (USD 4110)	1.682 (1.144–2.472)	0.008	2.028 (1.049–3.919)	0.035	1.206 (0.651–2.234)	0.552
RMB 30,000 to 49,999 (USD 4110~6850)	1.594 (0.994–2.557)	0.053	1.321 (0.556–3.141)	0.528	0.829 (0.368–1.865)	0.650
RMB 50,000 to 99,999 (USD 6851~13,701)	1.260 (0.777–2.045)	0.349	2.147 (0.941–4.898)	0.069	1.704 (0.789–3.682)	0.175
Reasons for living alone						
(Ref: Other)						
The absence of nearby children	1.697 (1.098–2.623)	0.017	1.236 (0.668–2.288)	0.500	0.728 (0.422–1.256)	0.254
Avoid inconveniencing their children	1.554 (1.061–2.276)	0.024	1.224 (0.704–2.129)	0.474	0.788 (0.478–1.198)	0.350
Self-rated health status	2.066 (1.794–2.379)	<0.001	5.408 (4.336–6.747)	<0.001	2.618 (2.164–3.166)	<0.001
Cognitive dysfunction	1.300 (0.905–1.867)	0.155	1.962 (1.243–3.096)	0.004	1.509 (1.050–2.169)	0.026
Activities of daily living	0.916 (0.804–1.045)	0.192	0.944 (0.797–1.117)	0.501	1.030 (0.908–1.168)	0.648
Emotional support	1.127 (0.998–1.273)	0.053	0.826 (0.693–0.984)	0.033	0.732 (0.629–0.853)	<0.001

Note: C1 represents the mild depression–sleep disturbance group; C2 represents the moderate depression–forcefulness group; C3 represents the major depression–loneliness loss group; OR = Odds ratio; 95%CI = 95% Confidence Interval.

**Table 3:** Univariate analysis of self-neglect among older people living alone.

Variable	Self-Neglect Score	*F*/*t*/*r*	*p* Value
Age (years)		30.788	<0.001
60–74	2.59 ± 1.335		
75–89	2.93 ± 1.416		
≥90	3.34 ± 1.302		
Gender		−0.879	0.380
Male	2.95 ± 1.418		
Female	3.01 ± 1.379		
Residence		80.265	<0.001
City	2.16 ± 1.426		
Town	3.20 ± 1.325		
Rural	3.16 ± 1.308		
Years of education		82.900	<0.001
0	3.28 ± 1.328		
1–6	2.88 ± 1.339		
≥7	2.08 ± 1.353		
Source of Livelihood		86.103	<0.001
Retirement pay	2.23 ± 1.358		
Family	3.23 ± 1.337		
Other	3.16 ± 1.309		
Marital status		3.214	0.040
Married	2.79 ± 1.290		
Widowed	2.99 ± 1.401		
Divorced/Unmarried	3.28 ± 1.434		
Cognitive function		7.690	<0.001
Cognitive dysfunction	3.59 ± 1.283		
Normal cognitive function	2.88 ± 1.386		
Distance to medical institutions (kilometers)		0.206	0.554
<5	2.98 ± 1.400		
≥5	3.03 ± 1.359		
Self-rated health status		0.230	<0.001
Activities of daily living		−0.051	0.030

**Table 4:** Correlation analysis between depression and self-neglect in older adults living alone.

Variable	1	2	3	4	5	6
1 Depression	1					
2 Mode of life	0.262 **	1				
3 Social communication neglect	0.100 **	0.180 **	1			
4 Medical care neglect	0.071 **	0.097 **	0.067 *	1		
5 Living condition neglect	0.158 **	0.095 **	0.023	0.067 *	1	
6 Self-neglect	0.306 **	0.743 **	0.367 **	0.450 **	0.557 **	1

Note: * represents *p* < 0.01; ** represents *p* < 0.001.

**Table 5:** Generalized linear regression of factors influencing self-neglect in older adults living alone.

Variable	B	SE	$\chi^{2}$ Value	0R (95%CI)	*p*
Depression profiles (Ref: C1 mild depression–sleep disturbance group)					
C2 moderate depression– forcefulness group	0.235	0.069	11.687	1.264 (1.105–1.446)	0.001
C3 major depression– loneliness loss group	0.713	0.101	49.830	2.040 (1.673–2.486)	<0.001
Age (Ref: 60–74)					
75–89	0.189	0.084	5.052	1.208 (1.024–1.423)	0.025
≥90	0.511	0.096	28.093	1.666 (1.380–2.012)	<0.001
Residence (Ref: City)					
Town	0.440	0.104	17.755	1.553 (1.265–1.906)	<0.001
Rural	0.426	0.103	17.204	1.531 (1.252–1.872)	<0.001
Years of education (Ref: ≥7)					
0	0.525	0.104	25.690	1.690 (1.380–2.070)	<0.001
1–6	0.364	0.101	12.955	1.439 (1.180–1.754)	<0.001
Sources of livelihood (Ref: Retirement pay)					
Family	0.384	0.098	15.376	1.468 (1.212–1.779)	<0.001
Other	0.372	0.101	13.599	1.450 (1.190–1.779)	<0.001
Self-rated health status	0.218	0.036	36.625	1.243 (1.159–1.334)	<0.001
Cognitive dysfunction	0.292	0.087	11.227	1.340 (1.129–1.589)	0.001

Note: OR = Odds ratio; 95%CI = 95% Confidence Interval.

## Data Availability

The dataset could be applied on the CLHLS website: https://opendata.pku.edu.cn/dataverse/CHADS (accessed on 21 September 2024).

## Supplementary Materials

The following supporting information can be downloaded at: https://www.mdpi.com/article/10.3390/healthcare13060676/s1, Table S1: General characteristics of elderly people living alone. Table S2: Differential characterization of potential profiles of depressive symptoms in older adults living alone.

## Abbreviations

The following abbreviations are used in this manuscript:

AIC	Akaike information criterion
aBIC	Sample size adjusted BIC
BIC	Bayesian information criterion
BLRT	Bootstrapped likelihood ratio test
CI	Confidence Interval
CLHLS	Chinese Longitudinal Healthy Longevity Survey
C1	Depression–sleep disturbance group
C2	Moderated depression–forcefulness group
C3	Major depression–loneliness loss group
LMR	Lo–Mendell–Rubin likelihood ratio test
LCA	Latent Class Analysis
LCM	Latent Class Model
LPA	Latent Profile Analysis
OR	Odds Ratio
