# Supplementary material for: The Relationship Between Depressive Symptoms and Self-Neglect in Chinese Older Adults Living Alone: A Latent Profile Analysis
Source: Healthcare (Basel). 2025 Mar 20;13(6):676. doi: 10.3390/healthcare13060676 (PMC11941949; doi:10.3390/healthcare13060676)
Supplement: Supplementary file 1 [file healthcare-13-00676-s001.zip › healthcare-3457520-supplementary.pdf]

Table S1. General characteristics of elderly people living alone (n=1822)

Variable	n/M	%/IQR
Age (years)		
60-74	309	17.0
75-89	1009	55.4
≥90	504	27.6
Gender		
Male	702	38.5
Female	1120	61.5
Residence		
City	346	19.0
Town	611	33.5
Rural	865	47.5
Years of education		
0	989	54.3
1-6	580	31.8
≥7	253	13.9
Source of Livelihood		
Retirement pay	411	22.6
Family	831	45.6
Other	580	31.8
Annual family income (RMB）		
<30000	1296	71.1
30,000 to 49,999	190	10.4
50,000 to 99,999	172	9.5
≥100,000	164	9.0
Marital status		
Married	172	9.5
Widowed	1582	86.8
Divorced/Unmarried	68	3.7
Reasons for living alone		
The absence of nearby children	413	22.7
Avoid inconveniencing their children	1245	68.3
Other	164	9.0
Self-rated health status		
Very good	219	12.0
Better	641	35.2
Fair	731	40.1
Worse	214	11.8
Very bad	17	0.9
Cognitive function		
Cognitive dysfunction	259	14.2
Normal cognitive function	1563	85.8
Activities of daily living, scores	18	0
Emotional support	2	2

Table S2. Differential characterization of potential profiles of depressive symptoms in older adults living alone.

Variable	C1 (n=535)	C2 (n=1006)	C3 (n=281)	χ^2/H	P
Age (years)				4.083	0.395
60-74	100（18.7）	161（16.0）	48（17.1）		
75-89	302（56.4）	551（54.8）	156（55.5）		
≥90	133（24.9）	294（29.2）	77（27.4）		
Gender				11.579	0.003
Male	229（42.8）	387（38.5）	86（30.6）		
Female	306（57.2）	619（61.5）	195（69.4）		
Residence				35.571	< 0.001
City	130（24.3）	189（18.8）	27（9.6）		
Town	145（27.1）	345（34.3）	121（43.1）		
Rural	260（50.6）	472（46.9）	133（47.3）		
Years of education				27.148	< 0.001
0	259（48.4）	549（54.6）	181（64.4）		
1-6	181（33.8）	317（31.5）	82（29.2）		
≥7	95（17.8）	140（13.9）	18（6.4）		
Source of livelihood				28.977	< 0.001
Retirement pay	150（28.1）	228（22.7）	33（11.7）		
Family	235（43.9）	452（44.9）	144（51.3）		
Other	150（28.0）	326（32.4）	104（37.0）		
Annual family income (RMB）				35.395	< 0.001
<30000	341（63.7）	729（72.5）	226（80.4）		
30,000 to 49,999	61（11.4）	113（11.2）	16（5.7）		
50,000 to 99,999	62（11.6）	86（8.5）	24（8.6）		
≥100,000	71（13.3）	78（7.8）	15（5.3）		
Marital status				11.382	0.023
Married	63（11.8）	87（8.6）	22（7.8）		
Widowed	456（85.2）	885（88.0）	241（85.8）		
Divorced/Unmarried	16（3.0）	34（3.4）	18（6.4）		
Reasons for living alone				12.909	0.012
The absence of nearby children	102（19.1）	238（23.7）	73（17.7）		
Avoid inconveniencing their children	372（69.5）	694（69.0）	179（63.7）		
Other	61（11.4）	74（7.4）	29（17.7）		
Cognitive function				28.711	< 0.001
Cognitive dysfunction	52（9.7）	141（14.0）	66（23.5）		
Normal cognitive function	483（90.3）	865（86.0）	215（76.5）		
Activities of daily living [M (IQR)], scores	18（0）	18（0）	18（0）	12.256	0.002
Emotional support [M (IQR)]	2（2）	2（2）	2（2）	23.357	< 0.001

Note: C1 represents the mild depression-sleep disturbance group; C2 represents the moderate depression-forcefulness group; and C3 represents the severe depression-loneliness loss group; M represents median; IQR represents interquartile range.
